# Dietary supplementation with sodium isobutyrate enhances growth performance and colonic barrier function in weaned piglets via microbiota-metabolite-host interactions

**DOI:** 10.1186/s40104-025-01310-w

**Published:** 2025-12-08

**Authors:** Xiuyu Fang, Zihan Chi, Zhengyi Wang, Xinlin Wang, Xingrui Qu, Shuang Zhang, Feng Gao, Baoming Shi, Xuan Zhao

**Affiliations:** 1 College of Animal Science and Technology, Southwest University, Chongqing, 400715 People’s Republic of China; 2 College of Animal Science and Technology, Northeast Agricultural University, Harbin, 150030 People’s Republic of China

**Keywords:** Colonic barrier function, Diarrhoea, Intestinal microbiota, Sodium isobutyrate, Weaned piglets

## Abstract

**Background:**

Weaning-induced diarrhoea and growth retardation in piglets are associated with impaired intestinal barrier function and decreased levels of colonic short-chain fatty acids (SCFAs). Although SCFA supplementation has been proposed to mitigate these issues, the efficacy and optimal dosage of sodium isobutyrate remain unclear.

**Results:**

We investigated the effects of sodium isobutyrate supplementation (500, 1,000, 2,000, and 4,000 mg/kg diet) on weaned piglets (Duroc × Landrace × Yorkshire, 28 d of age; *n* = 8). After a 28-d feeding trial, supplementation at 500–2,000 mg/kg significantly improved average daily gain and feed efficiency and reduced diarrhoea frequency, with maximal benefits observed at 1,000 mg/kg (*P* < 0.0001). Additionally, 500–1,000 mg/kg sodium isobutyrate supplementation increased the apparent digestibility of crude protein, organic matter, and crude fibre (*P* < 0.05). Serum biochemical parameters were unaffected, although secretory immunoglobulin A (SIgA) levels significantly increased upon supplementation with 500–1,000 mg/kg (*P* < 0.05). 16S rRNA gene sequencing indicated that sodium isobutyrate increased the abundance of beneficial colonic microbiota. The 1,000 mg/kg group presented the most pronounced effect, with a significant increase of the relative abundance of *Prevotella* and the greatest improvement in SCFA concentrations (*P* < 0.05). Metabolomics revealed elevated levels of colonic indole-3-lactic acid and 3-hydroxybutyrate upon supplementation with 1,000 mg/kg (*P* < 0.05). Transcriptomic analyses indicated activation of protein digestion and absorption pathways, and PI3K-Akt signalling, marked by *TSG-6* upregulation and the suppression of *ISG15* and *DDIT4* expression (*P* < 0.05). Supplementation with 1,000 mg/kg was associated with improved intestinal barrier-related markers, including reduced serum D-lactate, diamine oxidase, and lipopolysaccharide levels, increased tight junction protein expression; activation of G protein-coupled receptors; and inhibition of TLR4/MyD88/NF-κB signalling (*P* < 0.05), suggesting enhanced barrier function.

**Conclusions:**

In conclusion, dietary supplementation with 1,000 mg/kg sodium isobutyrate was associated with improved intestinal morphology, reduced serum permeability, increased expression of tight junction proteins, and enhanced immune function in weaned piglets, suggesting enhanced colonic barrier function and providing dosage guidance and mechanistic insights for future applications.

**Graphical Abstract:**

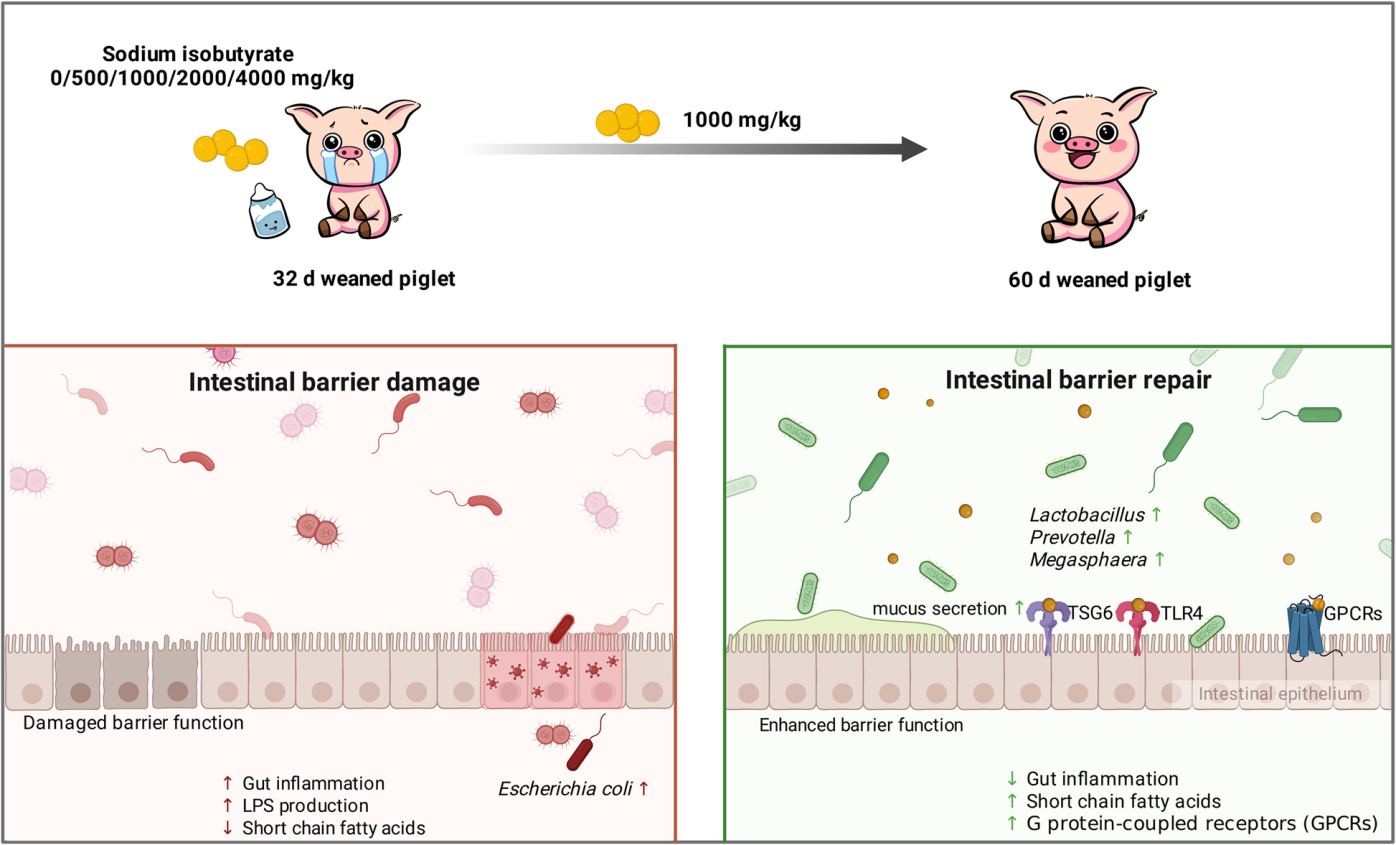

**Supplementary Information:**

The online version contains supplementary material available at 10.1186/s40104-025-01310-w.

## Introduction

Weaning piglets in the pig production industry at 3–4 weeks of age is a relatively stressful event occurring at a sensitive period in their life cycle. At this time, their intestinal development and immune system are not yet fully developed, and the piglets face multiple nutritional, physiological, and environmental challenges [1]. These weaning stress factors can induce dysbiosis in the gut microbiota, damage the intestinal barrier, and consequently lead to intestinal and systemic inflammation in piglets [2]. Additionally, this can result in reduced intake of feed, weight loss, and an increased incidence of diarrhoea. With the complete ban on antibiotic use in feed, finding a safe and reliable method to improve weaning injuries in piglets is an urgent problem that researchers need to address [3, 4].

Short-chain fatty acids (SCFAs) produced by the gut microbiota can regulate homeostasis of the intestinal microbial community [5] and play crucial roles in maintaining intestinal barrier function and alleviating intestinal inflammation [6]. Our previous research revealed that impaired intestinal barrier function and dysbiosis are often accompanied by a decrease in isobutyrate levels [7, 8]. Isobutyrate is a branched short-chain fatty acid (BSCFA). Unlike SCFAs derived from the decomposition of dietary fibre, such as acetate and butyrate, BSCFAs such as isobutyrate and isovalerate originate from protein metabolism [9]. Previous studies have shown that isobutyrate can effectively regulate the metabolic balance of liver lipids and alleviate atherosclerosis [10]. Other studies suggest that BSCFAs may be related to the regulation of host immune function [11].

Mechanistically, sodium isobutyrate may exert regulatory effects on host health by activating G-protein coupled receptors (GPCRs) and suppressing proinflammatory signalling pathways. As GPCRs are among the major receptors for SCFAs, they play important roles in modulating epithelial barrier function as well as mucosal and systemic immunity [6]. To verify these potential effects, evaluating reliable indicators of intestinal barrier integrity and inflammation is essential. Key serum biomarkers, such as D-lactate (D-LA), diamine oxidase (DAO), and lipopolysaccharide (LPS), have been widely recognized as indicators of intestinal barrier function [12]. When the intestinal barrier is disrupted, LPS derived from Gram-negative bacteria can enter the circulation, leading to endotoxaemia and systemic inflammation [13]. Moreover, the levels of inflammatory cytokines provide complementary information: IL-1β and TNF-α are classical proinflammatory mediators, whereas IL-10 serves as a key anti-inflammatory cytokine [14]. Beyond these functional readouts, molecular pathways and gene expression profiles can provide mechanistic insights. In particular, the PI3K-Akt signalling pathway is crucial for maintaining intestinal epithelial integrity and immune responses [15], while genes such as *TSG-6*, *DDIT4*, and *ISG15* are involved in inflammation resolution, stress adaptation, and innate immunity, respectively [16].

In addition, previous research by our team revealed that *Bacillus siamensis*, a bacterium capable of producing isobutyrate, can significantly improve intestinal barrier function in piglets and mice [17, 18] and strongly inhibits pathogenic bacteria (Figs. S1, S2 and Table S1). On the basis of the critical role of SCFAs in regulating intestinal health and our previous study results [8, 19], we hypothesized that sodium isobutyrate may improve intestinal barrier function and immunological function by activating GPCRs, suppressing proinflammatory signalling pathways and optimizing the intestinal microbiota and its metabolites, thereby alleviating weaning stress and improving the growth performance of piglets.

In summary, research on the direct addition of sodium isobutyrate to feed to alleviate weaning stress in piglets is very limited, and both the supplementation amount and mechanisms of action remain unclear. To address these gaps, the present study was designed to evaluate the effects and underlying mechanisms of dietary sodium isobutyrate supplementation in weaned piglets.

## Methods

### Animals and experimental design

A total of 40 weaned piglets (Duroc × Landrace × Yorkshire crossbred) aged 28 d were randomly allocated to 5 dietary treatment groups balanced for sex on the basis of initial body weight (*n* = 8 per group, one piglet per replicate). All the piglets were acclimated for 3 d with the basal diet prior to the 28-d experimental period, during which time they were fed diets supplemented with various levels of sodium isobutyrate. On the basis of the commonly applied sodium butyrate supplementation standards [20], the treatment groups received 0 mg/kg (control, CON), 500 mg/kg, 1,000 mg/kg, 2,000 mg/kg, and 4,000 mg/kg sodium isobutyrate (NaIB). The composition and nutritional adequacy of the diets adhered to the guidelines established by the National Research Council (2012) [21] and the Nutrient Requirements of Swine (2020) [22], as detailed in Table 1. Each piglet was housed individually in a temperature- and humidity-controlled metabolic crate (maintained at 26 ± 1 °C and 60%–70% relative humidity) to ensure accurate measurement of feed intake and performance metrics. The animals had unrestricted access to fresh water throughout the study. Prior to sampling, the animals were fasted for 12 h. On d 29, the piglets were humanely sacrificed by electrical stunning followed by exsanguination. Blood samples from experimental piglets were collected from the anterior vena cava using serum separation gel tubes. After standing at room temperature for 30 min, the samples were centrifuged at 3,000 × *g* for 10 min to separate the serum. The serum was transferred to sterile 1.5-mL EP tubes and stored at −80 °C for later analysis. After serum collection, the piglets were slaughtered, and tissue samples were taken. Small intestine and colon tissues were sectioned into 2 cm rings and fixed in 4% formaldehyde at 4 °C for histological analysis. A 3 mm × 1 mm colon sample was placed in 2.5% glutaraldehyde for electron microscopy. Colonic tissue and digesta were collected, stored in 5-mL cryovials, rapidly frozen in liquid nitrogen, and kept at −80 °C for molecular research.

**Table 1:** Composition and nutrient levels of the basal diets (as-fed basis), %

Ingredients	Content	Nutrient^2^	Content
Corn	45.24	Net energy, Mcal/kg	2.52
Hulled soybean meal	16.5	Lysine	1.25
Puffed corn	12.00	Methionine	0.37
Rice bran meal	2.50	Threonine	0.73
Whey powder	5.00	Tryptophan	0.22
Glucose	2.00	Crude protein	18.73
Whole Puffed Soya	9.00	Crude fat	3.93
Fish meal	3.00	Crude fiber	2.94
Soybean oil	1.20	Total calcium	0.74
Calcium dihydrogen phosphate	0.85	Total phosphorus	0.65
Limestone	0.98		
NaCl	0.4		
L-Lysine HCl	0.28		
DL-Methionine	0.05		
Premix^1^	1.00		
Total	100.00		

^1^Premix provided the following per kilogram of feed: vitamin A, 12,500 IU; vitamin D_3_, 2,800 IU; vitamin E, 30 IU; vitamin K_3_, 5 mg; vitamin B_1_, 1.5 mg; vitamin B_6_, 3 mg; vitamin B_12_, 40 μg; riboflavin, 15 mg; pantothenic acid, 15 mg; niacin, 40 mg; folic acid, 1 mg; biotin, 0.08 mg; choline chloride, 500 mg; Mn, 4 mg; Fe, 100 mg; Zn, 80 mg; Cu, 6 mg; I, 0.7 mg; Se, 0.48 mg

^2^Crude protein, crude fat, crude fiber, total calcium and total phosphorus values were obtained by analysis, while other values were calculated

### Growth performance and diarrhoea incidence

The feed intake of each piglet was recorded daily, and body weight was measured every 14 d to determine changes in weight, and these data were subsequently used to calculate the average daily feed intake (ADFI), average daily gain (ADG), and feed conversion ratio (FCR). FCR was calculated as the ratio of feed intake (kg) to body weight gain (kg) over the experimental period. Diarrhoea was monitored daily at 6:00 h in each group using a faecal scoring system (0 = normal, 1 = soft stool, 2 = pasty stool, 3 = watery stool), with scores ≥ 2 defined as diarrhoea [23]. Every 14 d, the rate of diarrhoea of each group of piglets was calculated, with the diarrhoea rate of each piglet defined as the number of diarrhoea events divided by 14.

### Nutrient digestibility

The dietary nutrient composition was analyzed in accordance with the Chinese National Standards. Crude protein (CP; GB/T 6432—2018) was measured using a Kjeltec 8400 apparatus (FOSS Inc., Minnesota, USA), with protein content calculated as nitrogen multiplied by 6.25. Ether extract (EE; GB/T 6433—2006) was determined using an ANKOMXT15 fat extractor (ANKOM Technology, USA). Neutral detergent fiber (NDF; GB/T 20806—2022) was analyzed with an ANKOM 200 fiber analyzer (ANKOM Technology, New York, USA). Calcium (GB/T 6436—2018) and total phosphorus (GB/T 6437—2018) contents were quantified via spectrophotometry (UV-2401, Shimadzu, Kyoto, Japan), and ash content was assessed following GB/T 6438—2007. Acid-insoluble ash (AIA) was determined by ashing. Apparent nutrient digestibility was assessed using AIA as an internal marker. Faecal samples were thawed, homogenized, dried at 65 °C, ground, and sieved (40 mesh). The digestibility of dry matter, CP, and EE was calculated from the nutrient and AIA contents in the diets and faeces according to the following equation: Apparent digestibility (%) = [1 − (N_feces × A_diet)/(N_diet × A_feces)] × 100, where N_diet and N_feces are the nutrient contents in the diet and faeces, respectively, and A_diet and A_feces are the AIA contents in the diet and faeces, respectively.

### Blood biochemical and antioxidant assays

Piglet serum biochemical parameters were measured at the Heilongjiang Provincial Hospital using an automated biochemical analyser (Hitachi 7180, Hitachi High-Technologies, Tokyo, Japan). Serum antioxidant parameters, including total antioxidant capacity (T-AOC; Cat. No. A015-2-1), total superoxide dismutase (T-SOD; Cat. No. A001-3-2), catalase (CAT; Cat. No. A007-1-1), glutathione peroxidase (GSH-Px; Cat. No. A005-1-2), and malondialdehyde (MDA; Cat. No. A003-1-1), were determined using commercial assay kits purchased from Nanjing Jiancheng Bioengineering Institute (Nanjing, China) according to the manufacturer’s instructions.

### Determination of intestinal barrier markers and inflammatory cytokines

The protein levels of D-LA (Cat. No. ML603756), DAO (Cat. No. MLO02413), LPS (Cat. No. ML002299), and the cytokines IL-1β (Cat. No. ML002302), IL-10 (Cat. No. ML002319), and TNF-α (Cat. No. ML002360) were quantified in colonic tissue homogenates using commercially available ELISA kits (Hnybio, Shanghai, China), according to the manufacturer’s instructions.

### DNA extraction

Genomic DNA was extracted from the microbial communities in piglet colonic digesta using the Omega Bio-tek DNA extraction kit (D4015, Vermont, USA) following the manufacturer’s protocol. The DNA was eluted with 50 μL of buffer and stored at −80 °C until downstream PCR analysis, which was conducted by LC Biotechnology Co., Ltd. (Hangzhou, China). The purity of the DNA isolates was assessed by measuring the OD_260_/OD_280_ ratio using a NanoDrop spectrophotometer, with a value between 1.8 and 2.0, indicating high purity.

### PCR amplification and 16S rRNA gene sequencing

The V3–V4 hypervariable regions of the bacterial 16S rRNA genes were amplified using the primers 341 F and 805R. Amplicon integrity and expected size were confirmed by electrophoresis on a 2% agarose gel. Sequencing adapters and sample-specific barcodes were ligated during library preparation. Library quality was assessed using an Agilent 2100 Bioanalyzer and quantification was performed with an Illumina library quantification kit. Only libraries exceeding a concentration threshold of 2 nmol/L were subjected to paired-end (2 × 250 bp) sequencing on an Illumina NovaSeq 6000 platform.

### Microbiota data analysis

Demultiplexing of paired-end reads was conducted using unique sample-specific barcodes, followed by barcode and primer trimming. Read pairs were then merged using FLASH. Quality filtering was performed with fqtrim (v0.94), and chimeric sequences were identified and excluded using Vsearch (v2.3.4). Amplicon sequence variants (ASVs) and the corresponding feature table were inferred using DADA2 (version 2019.7) [24]. To enable inter-sample comparisons, the ASV table was rarefied to a sequencing depth of 20,000 reads per sample. Alpha and beta diversity metrics were calculated in R using the phyloseq (v1.34.0) and vegan (v2.7–1) packages. β-Diversity was assessed on the basis of Bray–Curtis distances and tested for statistical significance using PERMANOVA with 999 permutations.

### Short-chain fatty acid quantification

SCFAs in the colonic digesta were quantified using gas chromatography (GC). Approximately 2 g of digesta was homogenized with 2 mL of ultrapure water and extracted at 4 °C for 48 h, after which 400 µL of crotonic acid-metaphosphoric acid deproteinizing buffer (25%, w/v) was added as an internal standard. After centrifugation at 13,000 × g for 10 min at 4 °C, the supernatant was filtered through a 0.22-µm aqueous membrane, and the centrifugation-filtration process was repeated once to ensure accuracy. A mixed stock standard solution was prepared with acetate, propionate, isobutyrate, butyrate, isovalerate, and valerate, serially diluted with deproteinizing buffer to five concentrations, and used to generate calibration curves by plotting the ratio of the SCFA peak area to that of the internal standard (Ar/As) against the corresponding concentration ratio (mr/ms). GC analysis was carried out with an HP-INNOWax capillary column (30.0 m × 320 µm × 0.5 µm; Agilent, California, USA) with previously published operating conditions [17].

### Protein expression analysis (western blotting)

Colonic tissue proteins were extracted using RIPA lysis buffer supplemented with phenylmethylsulfonyl fluoride (PMSF) to prevent proteolytic degradation. The total protein concentration was quantified through the bicinchoninic acid (BCA) method. Subsequently, proteins were resolved by sodium dodecyl sulfate–polyacrylamide gel electrophoresis (SDS-PAGE) based on molecular weight and then transferred onto polyvinylidene difluoride (PVDF) membranes. The membranes were blocked in TBST containing skimmed milk at 35 °C for 2 h and incubated overnight at 4 °C with the appropriate primary antibodies. After several TBST washes, the membranes were exposed to horseradish peroxidase (HRP)-conjugated goat anti-rabbit IgG (H + L) for 2 h. Chemiluminescence signals were developed using the BeyoECL Star Fluorescence Detection Kit and imaged using a UVItec gel documentation system. All chemical reagents for western blotting were obtained from Beyotime Biotechnology (Shanghai, China). Densitometric quantification of protein bands was carried out using ImageJ 6.0 software. Detailed information regarding antibodies, including source species, vendor, and dilution ratios, can be found in Table S3.

### Haematoxylin and eosin (H&E) staining

After euthanasia, tissue samples were collected from piglets. The small intestine was excised and sampled from three predefined sites: the duodenum (~ 5 cm distal to the pylorus), the mid-jejunum (the midpoint between the pylorus and the ileocecal valve), and the terminal ileum (~ 5–8 cm proximal to the ileocecal valve). Colon samples were collected from the distal colon, approximately 5 cm proximal to the anus, as a representative site of the large intestine. The small intestine and colon tissues were cut into ~ 2 cm rings, gently rinsed with cold physiological saline, and fixed in 4% paraformaldehyde at 4 °C for histological analysis. H&E staining was performed according to the method described by Zhao et al. [25]. Briefly, small intestine and colon samples were fixed in 4% paraformaldehyde for 24 h to preserve tissue integrity. Following fixation, the samples were gradually dehydrated using a graded series of ethanol concentrations and then rendered transparent with xylene. The cleared tissues were embedded in paraffin wax and maintained at 60 °C. Once solidified, the paraffin blocks were cut into thin sections approximately 4–5 µm thick using a microtome. To prepare the slides for staining, the sections were immersed in xylene to eliminate the paraffin and then rehydrated through a descending ethanol gradient. Haematoxylin staining was performed for 5 to 10 min to visualize the cell nuclei, after which the sections were rinsed under running water to remove excess stain. Eosin stain was applied for 2 to 5 min to highlight cytoplasmic structures. After a final rinse with water, the slides were dehydrated, cleared again in xylene, and sealed with coverslips. Microscopy images were acquired using a digital imaging system (Winmedic, Shandong, China) at a magnification of 200 × to document tissue morphology. The villus height and crypt depth of small intestinal samples were quantitatively analyzed using ImageJ software (v 6.0). For each sample, three random microscopic fields were selected for measurement. The villus height was defined as the distance from the villus apex to the interface between the villus and the crypt, while the crypt depth was measured from this interface down to the basal membrane. The mean values of villus height and crypt depth were then calculated for each piglet.

### Transmission electron microscopy (TEM) analysis

Colon samples were initially fixed in 2.5% glutaraldehyde and rinsed with phosphate-buffered saline (PBS). Postfixation was performed in PBS containing 1% osmium tetroxide for 2 h at ambient temperature, followed by additional washes with PBS and a graded dehydration process. The tissues were then embedded overnight. Polymerization proceeded in an oven at 60 °C for 48 h. Ultrathin sections (~ 80 nm) were prepared using a diamond knife mounted on an ultramicrotome. Imaging was conducted with a Hitachi HT7700 transmission electron microscope (Hitachi High-Technologies, Tokyo, Japan). For each pig, the brush border length in the colon was quantified by averaging measurements from three randomly selected visual fields.

### Gene expression analysis by RT-qPCR

Following sample collection, colonic tissues were rapidly frozen in liquid nitrogen, stored at −80 °C, and processed for RNA extraction at the earliest opportunity to minimize degradation. Total RNA was isolated using TRIzol reagent (Takara, Kyoto, Japan) and subsequently reverse-transcribed into complementary DNA (cDNA) with the PrimeScript™ RT Reagent Kit containing gDNA Eraser (Takara, Kyoto, Japan). The concentration and purity of RNA were determined using a NanoDrop ND-1000 spectrophotometer (NanoDrop Technologies, Wilmington, USA), ensuring that the OD_260_/OD_280_ ratio ranged between 1.8 and 2.0. RNA integrity was confirmed through agarose gel electrophoresis and further evaluated using an Agilent Bioanalyzer, with all samples exhibiting RNA integrity numbers (RIN) of 7 or higher. Messenger RNA (mRNA) expression levels were quantified using β-actin as the reference gene on an Applied Biosystems 7500 Real-Time PCR system, employing the SYBR Green Master Mix Kit (Takara, Kyoto, Japan). Cycle threshold (Ct) values were used to quantify gene expression, and the 2^−ΔΔCt^ method was applied to normalize expression levels to those of β-actin [8]. Primers were designed using the NCBI Primer-BLAST tool, with specificity verified by BLAST analysis and melt curve assessment. The expression of differentially expressed genes identified by transcriptome analysis, as well as that of tight junction (TJ)-related genes, was quantified. The primer sequences for *TSG-6*, *DDIT4*, *ISG15*, *ZO-1*, *OCLN*, and *CLDN1* are listed in Table S2.

### Colonic metabolite analysis

The analytical approach for profiling colonic metabolites followed previously established protocols [7, 8]. Differentially abundant metabolites were identified by integrating univariate and multivariate statistical analyses. Specifically, fold changes and t tests adjusted using the Benjamini-Hochberg (BH) method were employed to assess statistical significance (*P* < 0.05), while variable importance in projection (VIP) values derived from partial least squares discriminant analysis (PLS-DA) were used to evaluate discriminatory power. Metabolites were considered significantly differentially abundant if they met all of the following criteria: a fold change ≥ 1.5 or ≤ 0.67, *P* < 0.05, and a VIP score ≥ 1.

### RNA-seq analysis

Following collection, colonic tissue samples were rapidly snap-frozen in liquid nitrogen, stored at −80 °C, and processed for RNA extraction promptly to minimize degradation. Total RNA was extracted from the colon using TRIzol reagent (Invitrogen, California, USA) according to the manufacturer’s instructions. After sample collection, the colonic tissues were immediately snap-frozen in liquid nitrogen, stored at −80 °C, and used for RNA extraction as soon as possible to prevent degradation. The requirements for RNA quality, quantity, and integrity were consistent with those described above. Only RNA samples that met the criteria of high quality were selected for complementary DNA (cDNA) library preparation. Sequencing was carried out using 150 bp paired-end reads on an Illumina NovaSeq 6000 platform (LC Bio, Hangzhou, China). Transcript levels were measured using the fragments per kilobase of transcript per million mapped reads (FPKM) normalization method. Genes showing more than a twofold difference in expression, with statistical significance set at *P* < 0.05, were classified as differentially expressed. These gene sets were further annotated and analysed for biological function and pathway involvement using Kyoto Encyclopedia of Genes and Genomes (KEGG) enrichment tools.

### Statistical analysis

All statistical analyses were performed using GraphPad Prism 10.2 software (GraphPad Software Inc., California, USA; https://www.graphpad.com). Two-group comparisons were analyzed by two-tailed Student's unpaired t-tests, with statistical significance set at *P* < 0.05. For datasets with over three groups, we applied one-way analysis of variance (ANOVA) followed by Tukey's post hoc test, considering an adjusted *P*-value < 0.05 as significant. Before applying the parametric tests, the normality of the data distribution was assessed using the Shapiro–Wilk test, and homogeneity of variance was evaluated with Levene’s test. For 16S rRNA sequencing data, we used non-parametric methods: the Mann-Whitney U test for two groups and the Kruskal-Wallis test with Dunn's post hoc test for multiple groups. All the data are presented as the mean ± SEM.

## Results

### Effects of different levels of dietary sodium isobutyrate on the growth performance of and nutrient digestibility in weaned piglets

The ADG results indicate that supplementation with 500, 1,000, and 2,000 mg/kg sodium isobutyrate can significantly increase the daily weight gain of piglets from d 1 to d 14 (*P* < 0.05; Table 2), whereas from d 15 to d 28, supplementation with only 1,000 mg/kg sodium isobutyrate can significantly increase the daily weight gain of piglets (*P* < 0.05). The FCR results indicate that adding 500, 1,000, and 2,000 mg/kg sodium isobutyrate can significantly reduce the FCR in piglets and increase feed efficiency. Moreover, the addition of different concentrations of sodium isobutyrate significantly reduced the diarrhoea frequency from d 1 to d 14, whereas the addition of 500, 1,000, and 2,000 mg/kg sodium isobutyrate effectively reduced the diarrhoea frequency from d 15 to d 28 (*P* < 0.05). Furthermore, we examined nutrient digestibility in piglets. The results showed that supplementation with sodium isobutyrate can improve the digestibility of organic matter and crude protein and significantly increase the digestibility of crude fibre, with the most significant improvement in crude fibre digestibility observed upon supplementation with 1,000 mg/kg (*P* = 0.0011; Table 3). On the basis of various performance indicators, we determined that 1,000 mg/kg sodium isobutyrate was an appropriate dosage.

**Table 2:** Effects of different levels of sodium isobutyrate on growth performance and frequency of diarrhoea in weaned piglets (*n* = 8)

Item	Sodium isobutyrate, mg/kg					SEM	*P*-value
	0	500	1,000	2,000	4,000		
Initial weight, kg	7.57	7.53	7.50	7.47	7.45	0.07	0.988
1–14 d							
ADG, g	128^b^	175^a^	196^a^	167^a^	127^b^	6.80	< 0.001
ADFI, g	299^c^	360^ab^	381^a^	352^abc^	310^bc^	9.60	0.022
FCR	2.35^ab^	2.06^c^	1.95^c^	2.12^bc^	2.50^a^	0.05	< 0.001
Diarrhea frequency, %	34.52^a^	20.24^bc^	14.29^c^	16.67^c^	26.19^b^	3.70	< 0.001
15–28 d							
ADG, g	351^b^	361^ab^	400^a^	364^ab^	332^b^	6.70	0.016
ADFI, g	690	683	708	701	656	7.20	0.162
FCR	1.97^a^	1.89^ab^	1.77^b^	1.94^a^	1.99^a^	0.02	0.014
Diarrhea frequency, %	17.86^a^	5.95^b^	2.38^b^	7.14^b^	14.29^a^	2.90	< 0.001
1–28 d							
ADG, g	240^c^	268^b^	298^a^	265^b^	230^c^	7.30	< 0.001
ADFI, g	494^bc^	521^ab^	545^a^	527^a^	483^c^	5.60	< 0.001
FCR	2.07^ab^	1.95^c^	1.83^d^	1.99^bc^	2.11^a^	0.02	< 0.001

*ADG* Average daily gain, *ADFI* Average daily feed intake, *FCR* Feed conversion ratio

^a–c^Different letter superscripts in the same row represent significant differences (*P* < 0.05)

**Table 3:** Effects of different levels of sodium isobutyrate on nutrient digestibility of weaned piglet diets (*n* = 6)

Item	Sodium isobutyrate, mg/kg					SEM	*P*-value
	0	500	1,000	2,000	4,000		
GE	78.9^ab^	80.00^a^	80.30^a^	77.97^bc^	76.40^c^	0.35	< 0.001
OM	81.81^b^	84.10^a^	83.79^a^	81.85^b^	80.41^c^	0.32	< 0.001
EE	60.36	57.89	59.85	58.54	56.04	0.7	0.298
CP	70.94^b^	73.48^a^	72.82^a^	69.30^cd^	67.83^d^	0.53	< 0.001
CF	29.27^d^	41.74^ab^	48.25^a^	38.99^bc^	32.80^cd^	1.65	0.001

*GE* Gross energy, *OM* Organic matter, *EE* Ether extract, *CP* Crude protein, *CF* Crude fiber

^a–d^Different letter superscripts in the same row represent significant differences (*P* < 0.05)

### Effects of different levels of sodium isobutyrate in feed on serum biochemistry, immunoglobulin and antioxidant levels in weaned piglets

Serum biochemical results indicate that feeding different levels of sodium isobutyrate has little impact on biochemical-related indicators, with all indicators tending towards a steady state and no adverse effects on piglets (*P* > 0.05; Table 4). Testing of immunoglobulins A (IgA) and secretory IgA in the blood showed that it can enhance the expression of SIgA in the blood (*P* < 0.05; Table 5). Further tests on antioxidant indicators in the blood revealed that 1,000 mg/kg sodium isobutyrate can significantly increase the expression levels of GSH-Px and CAT (*P* < 0.05).

**Table 4:** Effects of different levels of sodium isobutyrate on blood biochemical indices of weaned piglets (*n* = 6)

Item, mmol/L	Sodium isobutyrate, mg/kg					SEM	*P*-value
	0	500	1,000	2,000	4,000		
BUN	4.04	4.07	4.38	4.10	3.76	0.17	0.860
CREA	78.13	69.02	70.02	75.72	63.67	1.79	0.071
ALT	60.17	61.00	55.17	67.00	55.17	2.63	0.622
AST	112.00	101.00	107.33	101.50	111.50	9.05	0.993
GLU	5.09	5.02	4.42	4.76	4.08	0.15	0.158
TBA	43.07	35.75	54.98	38.57	28.93	5.38	0.656
TBIL	0.35	0.23	0.58	0.51	0.36	0.06	0.418
TP	55.17	52.83	48.90	56.22	56.40	1.14	0.197
ALB	32.15	29.93	29.03	32.93	30.23	0.70	0.378
GLOB	23.02	22.90	21.53	22.67	26.17	0.64	0.148
TG	0.68	0.50	0.45	0.69	0.65	0.05	0.421
CHOL	1.99	1.95	1.93	2.17	2.19	0.07	0.608
HDL	0.83	0.83	0.79	0.87	0.87	0.02	0.847
LDL	1.12	1.12	1.24	1.15	1.25	0.04	0.639

*BUN *Blood urea nitrogen, *CREA *Creatinine, *ALT *Alanine aminotransferase, *AST *Aspartate aminotransferase, *TBIL *Total bilirubin, *GLU *Glucose, *TBA *Total bile acid, *TP *Total protein, *ALB *Albumin, *GLOB *Globulin, *TG *Triglycerides, *CHOL *Total cholesterol, *HDL *High-density lipoprotein, *LDL *Low-density lipoprotein

**Table 5:** Effects of different levels of sodium isobutyrate on serum immunological and antioxidant indices of weaned piglets (*n* = 6)

Item	Sodium isobutyrate, mg/kg					SEM	*P*-value
	0	500	1,000	2,000	4,000		
IgA, μg/mL	519.05	469.27	470.34	493.52	557.76	13.7	0.229
IgM, mg/mL	12.67	10.20	10.81	12.15	12.67	0.50	0.404
IgG, μg/mL	13.14	10.05	10.11	10.41	11.95	0.47	0.143
SIgA, μg/mL	26.32^c^	34.16^ab^	38.76^a^	28.5^bc^	26.36^c^	1.37	0.005
GSH-Px, U/mL	491.91^b^	522.13^b^	703.55^a^	609.93^ab^	516.60^b^	26.20	0.038
CAT, U/mL	7.59^b^	10.59^ab^	14.94^a^	12.92^ab^	7.93^b^	0.92	0.036
MDA, nmol/mL	4.30	2.98	4.13	3.74	3.15	0.20	0.14
T-SOD, U/mL	18.16	15.13	20.31	16.47	14.81	0.85	0.21
T-AOC, U/mL	5.23	5.51	6.13	6.90	4.98	0.60	0.864

^a–c^Different letter superscripts in the same row represent significant differences (*P* < 0.05)

### Effects of different levels of sodium isobutyrate in feed on intestinal microbiota

**Fig. 1:**
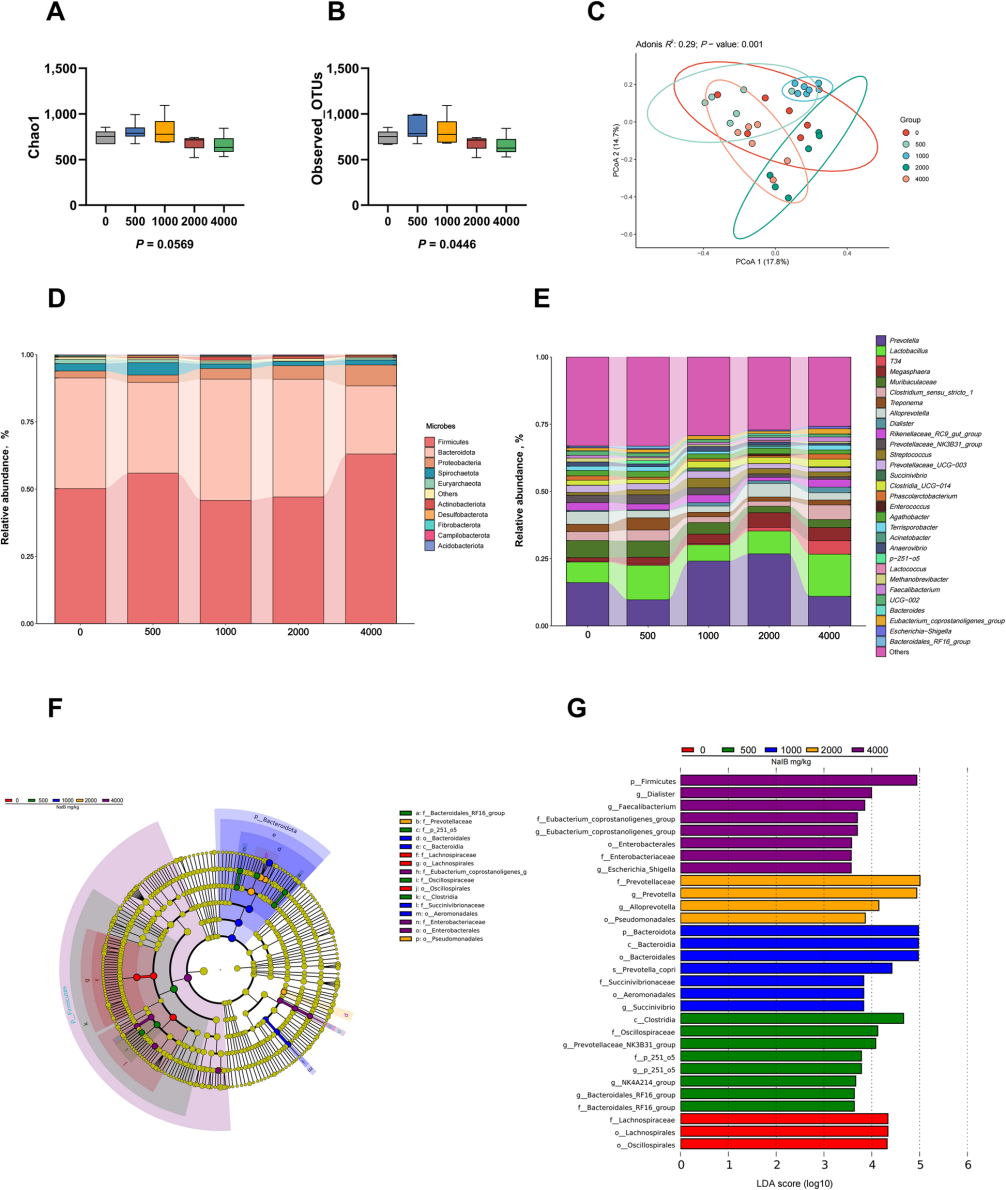
Effect of different levels of sodium isobutyrate on the colonic microbiota of weaned piglets. **A** and **B** Effect of different levels of sodium isobutyrate on the α diversity of the colonic microbiota of weaned piglets. **C** Effect of different levels of sodium isobutyrate on the β diversity of the colonic microbiota of weaned piglets. **D** Effect of different levels of sodium isobutyrate on the phylum level of colonic microbiota in weaned piglets. **E** Effects of different levels of sodium isobutyrate on colonic microbiota genus levels in weaned piglets. **F** and **G** Analysis of differences in colonic microbiota composition of weaned piglets by different levels of sodium isobutyrate. *n* = 6

We used 16S rRNA gene sequencing to detect the effect of adding different levels of sodium isobutyrate on the intestinal microbiota of piglets. The Chao1 index, which estimates species richness based on the number of rare operational taxonomic units (OTUs), and the Observed_OTUs index, representing the actual count of detected OTUs, were used to assess α-diversity. Results indicated that adding 1,000 mg/kg sodium isobutyrate to the diet did not significantly alter the levels of Chao1 and Observed_OTUs in piglets (Fig. 1A and B). PCoA analysis showed that significant differences were observed among all groups except between the control group and the 500 mg/kg sodium isobutyrate group (*P* < 0.05, Fig. 1C and Table S4). Analysis of the phylum level of intestinal microbiota in piglets showed that sodium isobutyrate at 1,000 and 2,000 mg/kg can increase the relative abundance of Fimicutes and decrease the relative abundance of Bacteroidota (Fig. 1D). Figure 1E shows the top 30 dominant bacterial genera in terms of abundance and percentage distribution for each treatment group, respectively. The dominant bacterial genera in the piglet colon were *Prevotella*, *Lactobacillus*, *Megasphaera*, *Muribaculaceae*, *Clostridium_sensu_stricto_1*, *Alloprevotella*, *Rikenellaceae_RC9_gut_group *and* Prevotellaceae_NK3B31_group*, among others. LEfSe analysis identified microbial taxa that showed significant differences among groups (Fig. 1F and G). At 500 mg/kg, the relative abundance of *Ruminococcaceae_NK4A214_group*, *Lachnospiraceae_RF16_group*, and *Prevotellaceae_NF3B31 _group* was increased. At 1,000 mg/kg, enrichment of *Prevotella_copri* and *Succinatvibrio* was observed. Supplementation with 2,000 mg/kg elevated *Prevotella* and *Alloprevotella*, while 4,000 mg/kg significantly increased the abundance of *Faecalibacterium* and the *Eubacterium_ coprostanoligenes_group*, among others (*P* < 0.05).

### Effects of different levels of sodium isobutyrate in feed additives on SCFAs and the expression of GPCRs in the colon

**Fig. 2:**
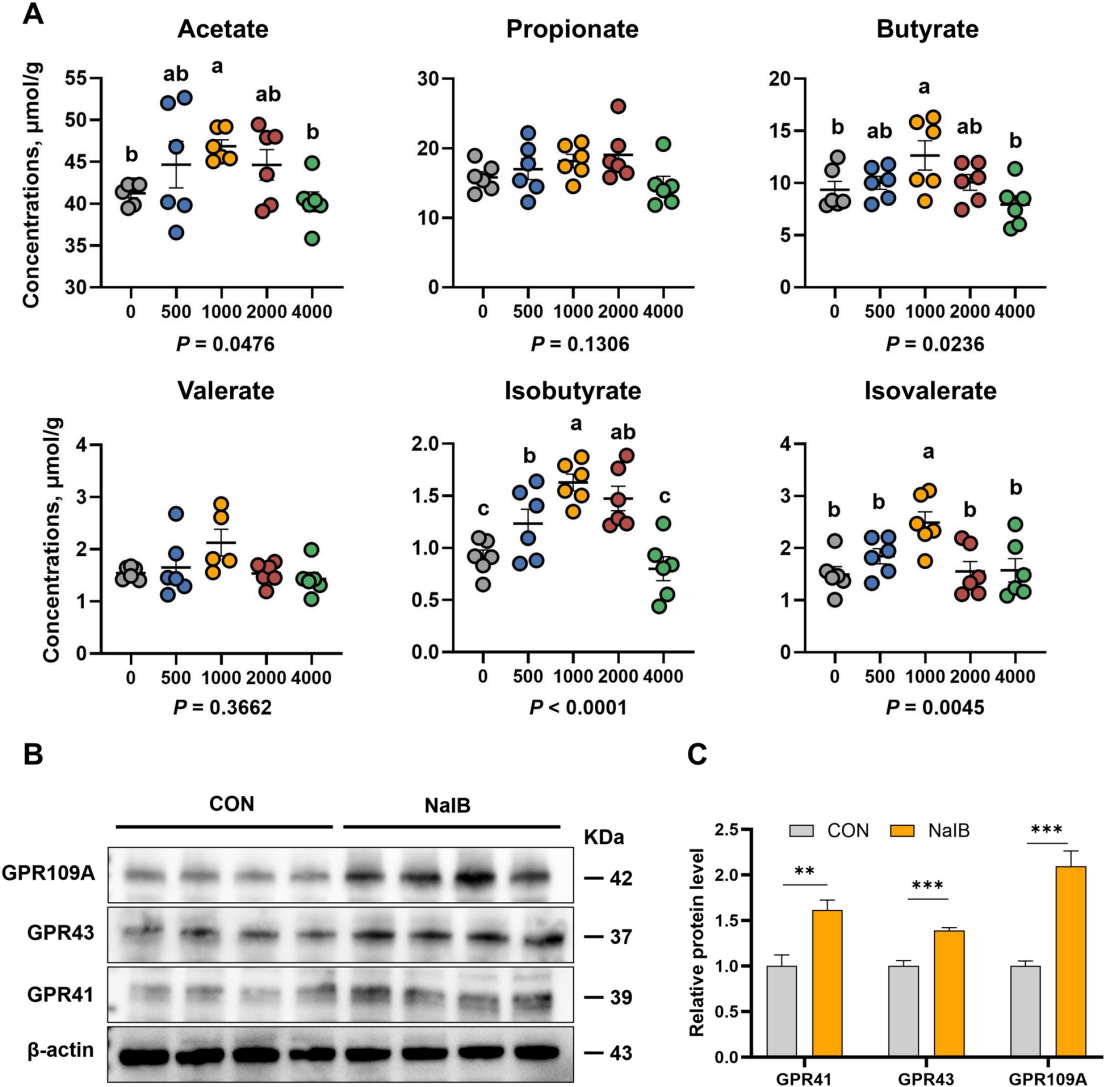
Effects of different levels of sodium isobutyrate on colonic short-chain fatty acids and G-protein-coupled receptors in weaned piglets. **A** Effects of different levels of sodium isobutyrate on colonic short-chain fatty acids (*n* = 6). ^a^^–^^c^Different letters represent significant differences (*P* < 0.05). **B** and **C** Effects of sodium isobutyrate on colonic G-protein-coupled receptors in weaned piglets (*n* = 4). ^*^Represents a significant difference between groups (*P* < 0.05); ^*^P < 0.05, ^**^P < 0.01, ^***^P < 0.001

We detected the SCFA levels in piglets' colons and found that at the dietary addition level of 1,000 mg/kg sodium isobutyrate, the levels of each SCFAs increased to varying degrees. Compared with the CON group, acetate, butyrate, isobutyrate, valerate, and isovalerate at 1,000 mg/kg all showed significant increases (*P* < 0.05; Fig. 2A). Adding 2,000 mg/kg and 4,000 mg/kg of sodium isobutyrate did not further increase the SCFA levels in the piglets' colons. Surprisingly, the isobutyrate levels in the piglets' colons did not increase with the higher amounts of sodium isobutyrate in the feed; the highest level of isobutyrate was observed when 1,000 mg/kg of sodium isobutyrate was added to the diet (*P* = 0.0007). One of the important pathways through which SCFAs regulate intestinal barrier function is by modulating GPCRs. Therefore, we tested the control group and the sodium isobutyrate group (1,000 mg/kg), and the results indicated that the expression levels of GPR41 (*P* = 0.0096), GPR43 (*P* = 0.0009), and GPR109A (*P* = 0.0009) were all significantly higher relative to the control group (Fig. 2B and C).

### Effects of sodium isobutyrate in feed on intestinal barrier function

**Fig. 3:**
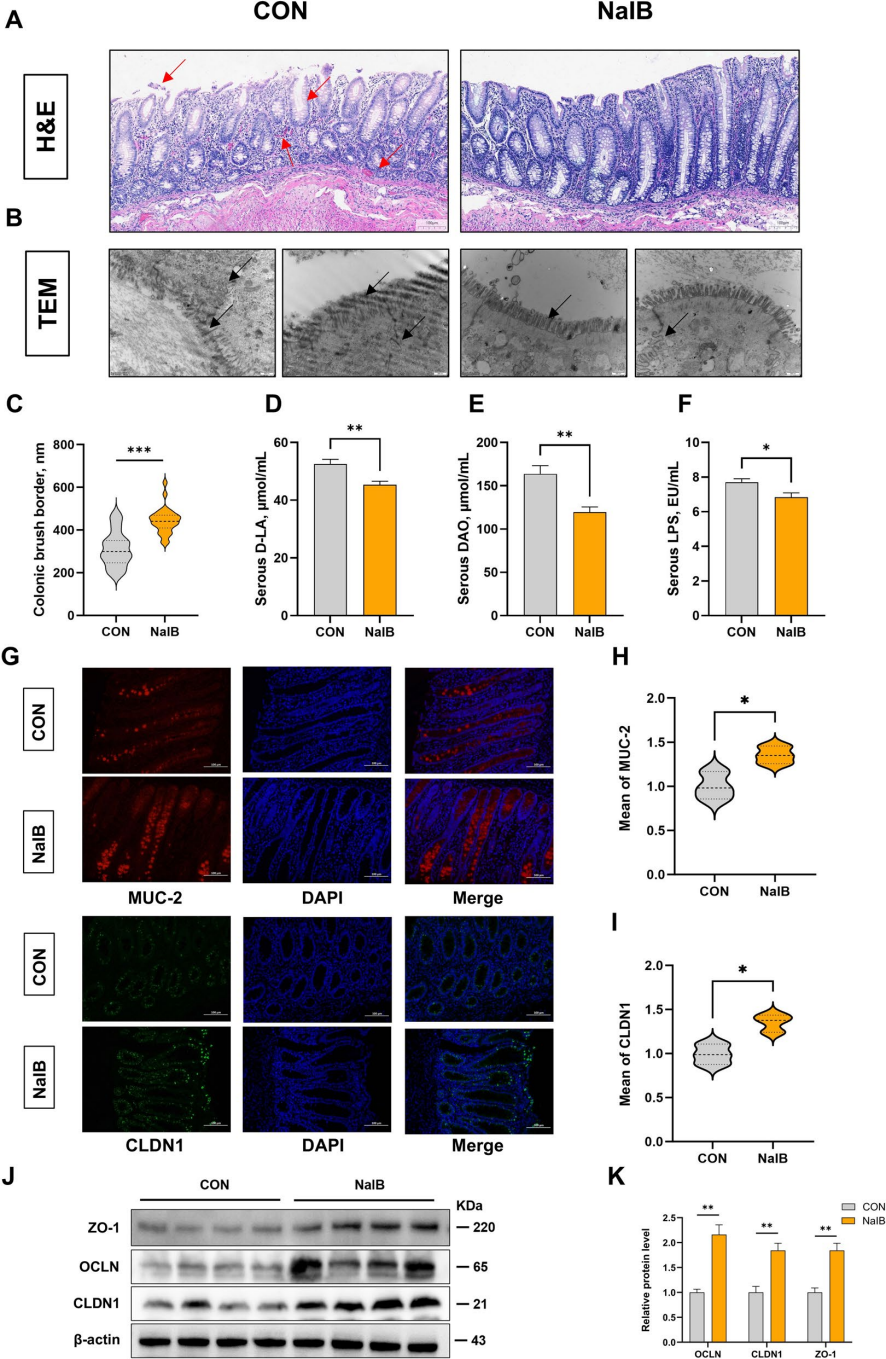
Effects of sodium isobutyrate on the colonic barrier function of weaned piglets. **A** Morphology of colon tissue sections was observed using hematoxylin and eosin staining (*n* = 6).** B** Transmission electron microscopy observation of colon ultrastructure (*n* = 6). **C** Measurement of colonic brush border (*n* = 6). **D**–**F** Determination of serum D-lactate, diamine oxidase and lipopolysaccharide in weaned piglets (*n* = 6). **G**–**I** Protein expression levels of colonic CLDN1 and MUC-2 were examined using immunofluorescence (*n* = 3). **J** and **K** Colonic tight junction protein expression levels (*n* = 4). ^*^Represents a significant difference between groups (*P* < 0.05); ^*^P < 0.05, ^**^P < 0.01, ^***^P < 0.001

Observation and measurement of the morphology of piglet small intestine showed that sodium isobutyrate can increase the ratio of jejunal villi to crypts, significantly promoting the ratio of ileal villi to crypts (*P* < 0.05; Table 6). Further, we conducted research on its mechanism of action. We compared the colonic barrier function between the control group and the sodium isobutyrate group. H&E staining revealed inflammatory cell infiltration in the lamina propria, crypt damage, and marked epithelial cell shedding in the control group, whereas sodium isobutyrate treatment significantly alleviated these pathological changes (Fig. 3A). TEM results revealed that the brush border length of the colon in the control group was shorter than that in the sodium isobutyrate group, with a significant difference observed (*P* < 0.0001; Fig. 3B and C). Moreover, the TJs in the sodium isobutyrate-treated group were clearly visible and well-defined, indicating improved mucosal barrier integrity. Further, we examined serum D-lactate, diamine oxidase, and LPS levels (Fig. 3D–F). Results showed that the sodium isobutyrate group significantly reduced the serum content of D-LA (*P* = 0.0057), DAO (*P* = 0.0029), and LPS (*P* = 0.0206). Subsequently, we measured the protein expression levels of TJ and mucin-associated markers in the colon (Fig. 3G–K). The results showed that the expression levels of ZO-1 (*P* = 0.0028), OCLN (*P* = 0.0014), CLDN1 (*P* = 0.0038), and MUC-2 (*P* = 0.0305) were significantly increased in the 1,000 mg/kg sodium isobutyrate-treated group compared with the control group.

**Table 6:** Effect of different levels of sodium isobutyrate on the morphology of the small intestine in weaned piglets (*n* = 6)

Item	Sodium isobutyrate, mg/kg					SEM	*P*-value
	0	500	1,000	2,000	4,000		
Duodenum							
Villus height, μm	218.31	201.03	192.63	198.53	203.07	4.42	0.525
Crypt depth, μm	97.53	86.38	88.24	86.84	111.65	4.55	0.343
V/C	2.29	2.38	2.30	2.27	1.97	0.09	0.612
Jejunum							
Villus height, μm	183.49	212.63	204.15	175.47	198.73	5.87	0.276
Crypt depth, μm	80.35	81.47	78.47	76.95	99.96	3.79	0.292
V/C	2.25^ab^	2.76^a^	2.85^a^	2.95^a^	1.88^b^	0.13	0.041
Ileum							
Villus height, μm	152.80	197.89	189.43	181.49	167.43	5.78	0.097
Crypt depth, μm	72.21	70.14	64.82	88.31	83.93	3.68	0.237
V/C	2.16^b^	3.27^a^	3.13^a^	2.18^b^	2.16^b^	0.15	0.015

^a,b^Different letter superscripts in the same row represent significant differences (*P* < 0.05)

### Effects of sodium isobutyrate to the feed on the colonic metabolites of piglets

**Fig. 4:**
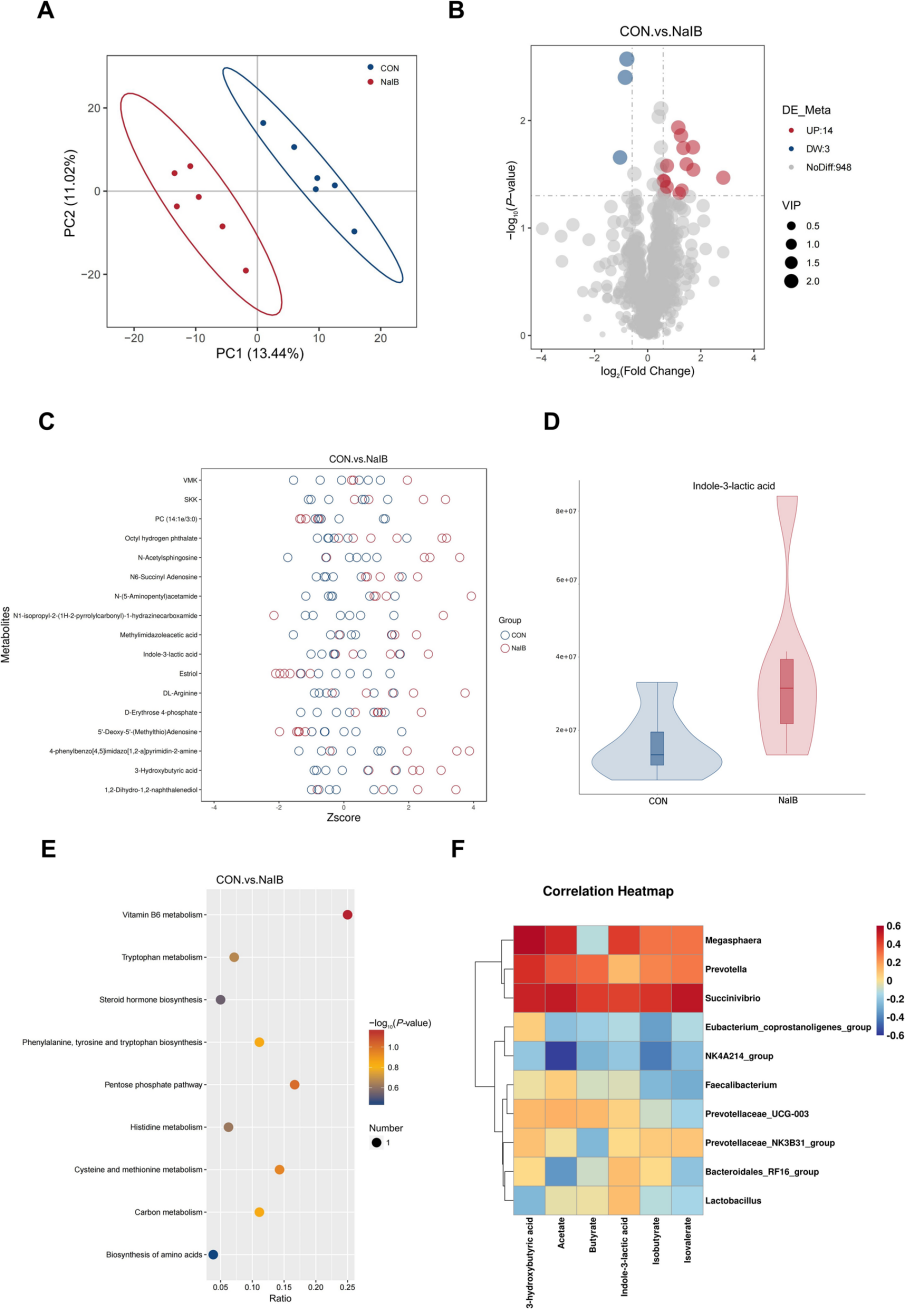
Effect of sodium isobutyrate on colonic metabolites in weaned piglets. **A** Partial least squares discriminant analysis of colonic metabolites in weaned piglet. **B** and **C** Analysis of differences in colonic metabolite composition of weaned piglets. **D** Relative concentration of indole-3-lactic acid. **E** Functional analysis on colonic differential metabolites in weaned piglets. **F** Probiotics and metabolites correlation analysis. *n* = 6

We further performed PLS-DA on the control and 1,000 mg/kg sodium isobutyrate groups and found a significant separation between the two (*P* < 0.05; Fig. 4A). Further we analysed the differential metabolites between the two and the results showed that the addition of sodium isobutyrate upregulated 14 differential metabolites and decreased 3 differential metabolites (*P* < 0.05; Fig. 4B). These differential metabolites were analysed and the metabolites found to be significantly elevated were indole-3-lactic acid, 3-hydroxybutyric acid, N-acetaylsphingosine, methylimidazoleacetic acid and DL- arginine, etc., with significant decreases in 5´-Deoxy-5´-(methylthio) adenosine and estriol, among others (*P* < 0.05; Fig. 4C and D). Further KEGG analysis revealed that the metabolite functions were mainly enriched in signalling pathways such as vitamin B_6_ metabolism, typtophan metabolism (Fig. 4E). Further we performed correlation analyses of the gut microbiota and metabolome, which showed a positive correlation between indole-3-lactic acid and the abundance of putative beneficial taxa such as *Lactobacillus*, a positive correlation between the concentration of 3-hydroxybutyric acid and the abundance of *Prevotella*, and a positive correlation between the concentration of SCFAs and the relative abundance of some putative beneficial taxa (Fig. 4F).

### Effects of the addition of sodium isobutyrate to the feed on the colon transcriptome

**Fig. 5:**
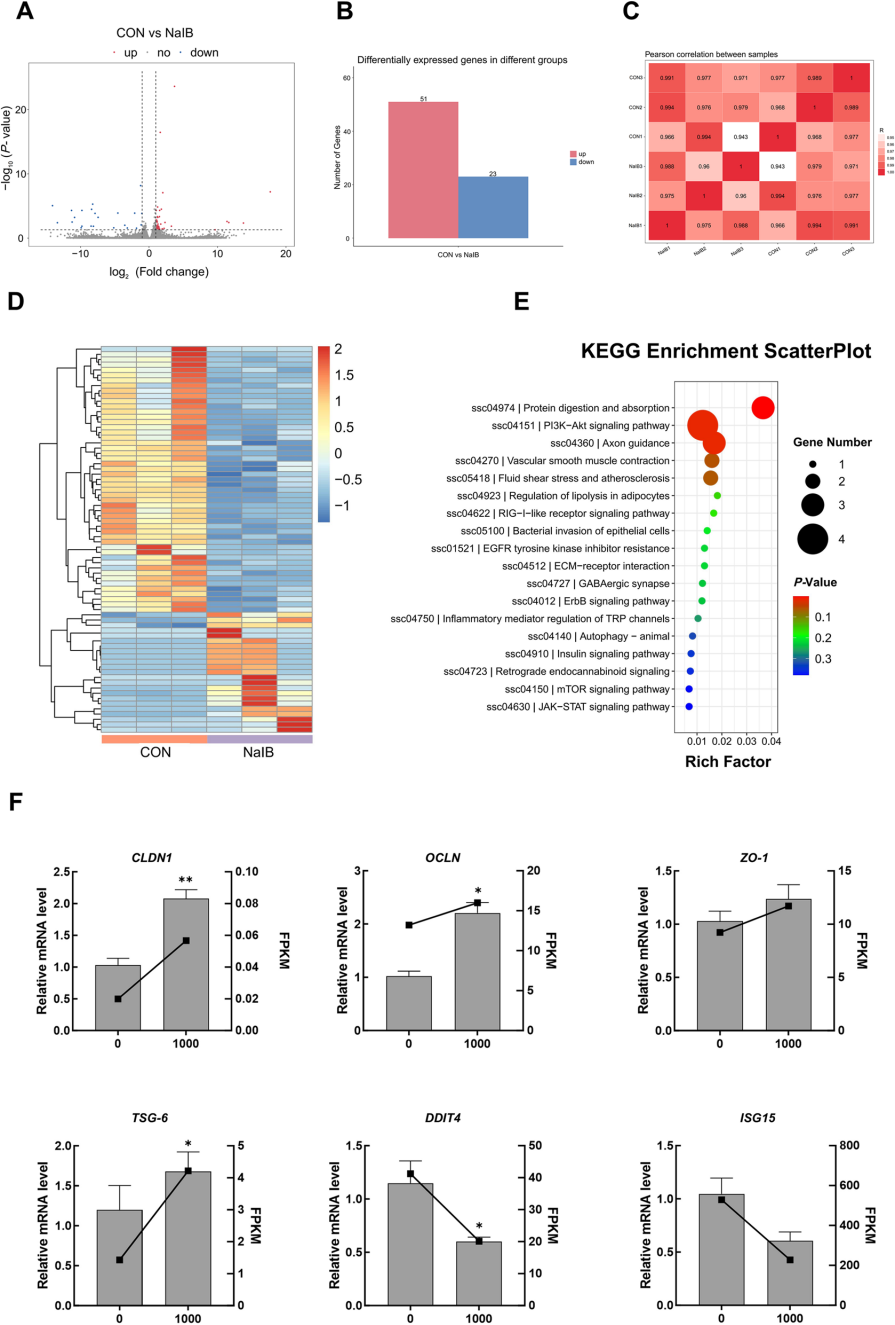
Effects of sodium isobutyrate on the colonic transcriptome of weaned piglets. **A** and **B** Changes in gene expression in the piglet colon. **C** Pearson correlation between samples. **D** Heat map of differential genes. **E** Differential gene enrichment kyoto encyclopedia of genes and genomes pathway map. **F** Differential gene transcriptome and PCR relative expression. *n* = 6. ^*^Represents a significant difference between groups (*P* < 0.05); ^*^P < 0.05, ^**^P < 0.01

Transcriptome analysis of the colon revealed that 1,000 mg/kg sodium isobutyrate supplementation was associated with significant changes in gene expression, with 51 genes upregulated and 23 genes downregulated (*P* < 0.05; Fig. 5A and B). KEGG pathway enrichment showed that these differentially expressed genes were mainly linked to protein digestion and absorption, PI3K-Akt signaling, mTOR signaling, JAK-STAT signaling, and other pathways (Fig. 5C–E). Differential expression analysis and PCR validation of representative genes further revealed that colonic mRNA levels of *CLDN1* and *OCLN* were significantly higher in piglets receiving sodium isobutyrate (*P* < 0.05), while *ZO-1* was correlated with a non-significant increasing trend. Moreover, supplementation with sodium isobutyrate was associated with a significant increase in the expression of *TSG-6* (*P* < 0.05), while the expression levels of *DDIT4* and *ISG15* were significantly decreased (*P* < 0.05; Fig. 5F).

### Effects of the addition of sodium isobutyrate to the feed on the TLR4/MyD88/NF-κB signalling pathway and inflammatory factors in the colon

**Fig. 6:**
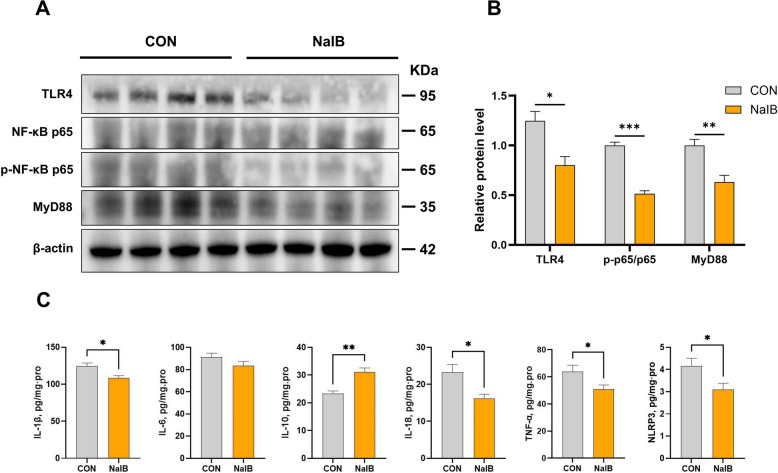
Effects of sodium isobutyrate on TLR4/MyD88/NF-κB signaling pathway and cytokines in the colon of weaned piglets. **A** and **B** Relative protein expression of TLR4/MyD88/NF-κB (*n* = 4). **C** Protein expression of cytokines (*n* = 6). ^*^Represents a significant difference between groups (*P* < 0.05); ^*^P < 0.05, ^**^P < 0.01, ^***^P < 0.001

SCFAs are known to interact with proinflammatory signalling pathways and exert anti-inflammatory effects. Therefore, we investigated whether sodium isobutyrate supplementation was associated with changes in the TLR4/MyD88/NF-κB pathway in the colons of weaned piglets. As shown in Fig. 6A and B, compared with the control treatment, supplementation with 1,000 mg/kg sodium isobutyrate was associated with lower protein expression of TLR4 (*P* = 0.0132) and MyD88 (*P* = 0.0068) and a lower p-p65/p65 ratio (*P* < 0.0001) in the colon. Consistent with these findings, the expression of cytokines and inflammasome-related factors in the colon also significantly changed: compared with the control treatment, sodium isobutyrate supplementation was associated with decreased expression of IL-1β (*P* = 0.0114), IL-18 (*P* = 0.0145), TNF-α (*P* = 0.0449), and NLRP3 (*P* = 0.0377), while the expression of IL-10 (*P* = 0.0018) significantly increased (Fig. 6C).

## Discussion

During weaning, piglets frequently experience diarrhoea, intestinal barrier damage, microbiota dysbiosis, and growth retardation due to incomplete intestinal development, which occasionally lead to severe mortality [2, 26]. Identifying strategies to mitigate postweaning growth impairment and intestinal barrier dysfunction remains a prominent research focus in animal husbandry [27]. With the global ban on antibiotic feed additives, exploring effective alternatives has become increasingly critical. Extensive research has demonstrated that SCFAs significantly modulate the gut microbiota and maintain intestinal barrier integrity [6, 28, 29]. Dietary supplementation with SCFAs such as acetate, butyrate, or sodium butyrate markedly enhances growth performance in weaned piglets [30, 31]. However, studies concerning isobutyrate remain limited. Unlike typical SCFAs produced via dietary fibre fermentation, isobutyrate is derived primarily from the microbial degradation of the amino acids in proteins, particularly valine [32]. The literature indicates that isobutyrate plays substantial roles in preventing atherosclerosis and regulating lipid metabolism and hepatic metabolic function [10, 33, 34]; nevertheless, its impact on colonic barrier function remains inadequately explored. Additionally, key questions, such as whether isobutyrate alleviates postweaning diarrhoea in piglets and the optimal dietary dosage, remain unanswered. Given the poor palatability and volatility associated with direct isobutyric acid supplementation, sodium isobutyrate was employed as a dietary additive in the present study. Therefore, to determine the optimal dosage and potential mechanisms for the application of sodium isobutyrate to weaned piglets, we carried out the following studies.

Postweaning diarrhoea is closely linked to impaired growth performance in piglets. In our study, sodium isobutyrate supplementation improved growth outcomes and reduced diarrhoea incidence in a dose-dependent manner, with the most consistent benefits observed at 1,000 mg/kg. Notably, excessive supplementation (4,000 mg/kg) failed to confer advantages and even impaired growth, likely due to reduced palatability and feed intake. Similar dose-sensitive responses have been reported with other organic acids and SCFAs, where excessive fermentation products may exacerbate gut dysfunction [31, 35]. These findings emphasize that sodium isobutyrate, like other dietary additives, exerts beneficial effects only within an optimal dosage range [36], which in this case appears to be near 1,000 mg/kg, which is consistent with the recommended levels of sodium butyrate [30, 37].

Apparent nutrient digestibility is a key indicator of feed utilization efficiency and intestinal health [38]. Previous studies have shown that organic acids can increase nutrient digestibility in livestock [39], but little is known about the effects of sodium isobutyrate in piglets. Our findings suggest that sodium isobutyrate may improve the digestibility of crude protein, organic matter, and fibre, likely by promoting a more favourable microbial composition that facilitates fermentation. In particular, the enrichment of fibre-degrading genera such as *Prevotella* provides a plausible explanation for the observed improvements in fibre utilization [40]. Conversely, excessive supplementation appeared to compromise energy digestibility, highlighting the dose-dependent nature of the effects of sodium isobutyrate and the need for careful dosage control. Interestingly, no significant changes were observed in crude fat digestibility, suggesting that sodium isobutyrate may have a limited influence on lipid metabolism under the current experimental conditions, which could be shaped by factors such as the dietary fat source and composition.

To accurately assess the physiological health and nutritional metabolism of weaned piglets fed sodium isobutyrate, we measured serum biochemical parameters [41]. No significant alterations in serum biochemical parameters, including BUN, CREA, ALT, AST, TBIL, GLU, TBA, TP, ALB, GLOB, TG, CHOL, HDL, and LDL, were observed in piglets supplemented with different dosages of sodium isobutyrate. These results indicate that sodium isobutyrate did not adversely affect hepatic or renal function.

Postweaning piglets have incompletely developed immune barrier functions, and serum immunoglobulins are critical indicators of piglet immunity [42]. Our results revealed that dietary sodium isobutyrate supplementation significantly increased serum SIgA concentrations. SIgA is vital for mucosal immunity and prevents pathogen adhesion, neutralizes intestinal toxins, and maintains microbial balance and immune homeostasis in the gut [43]. Furthermore, antioxidant capacity is another crucial indicator of the health status of weaned piglets. GSH-Px protects hosts from oxidative stress and regulates overall health, whereas CAT decomposes hydrogen peroxide to prevent oxidative cell damage and enhance immunity [44]. We found that piglets receiving 1,000 mg/kg sodium isobutyrate supplementation exhibited significantly elevated serum GSH-Px and CAT activities, reflecting beneficial effects on host antioxidant capacity.

Intestinal morphology is a critical indicator of digestive capacity and gut health [45]. In particular, small intestinal integrity is widely regarded as a hallmark of healthy growth in animals. Villus length is closely linked to nutrient absorption capacity, whereas crypt depth reflects stem cell activity and function [46]. Our findings suggest that 1,000 mg/kg sodium isobutyrate promotes mucosal development in key regions of the small intestine, enhancing nutrient absorption. Specifically, the increased villus-to-crypt ratio in the jejunum and ileum indicates improved intestinal structure, suggesting better intestinal function [47]. No significant changes were observed in the duodenum, indicating that the effects of sodium isobutyrate are more pronounced in the distal small intestine. These results underscore the potential of sodium isobutyrate to improve intestinal morphology and function, especially in the jejunum and ileum, which are crucial for digestion and nutrient absorption in weaned piglets. Additionally, approximately 95% of SCFAs, which are produced primarily in the caecum and colon, are absorbed and metabolized at the colonic brush border, provide essential energy to colonocytes and support microvillus development and barrier function [48, 49]. Conversely, colonic injury can severely disrupt brush border structures and impair intestinal health [50]. Consistent with previous studies showing that SCFAs improve intestinal morphology [51, 52], 500–2,000 mg/kg sodium isobutyrate supplementation appears to mitigate weaning-associated intestinal damage, likely by supporting epithelial renewal and maintaining mucosal integrity. These findings suggest that sodium isobutyrate plays a protective role in maintaining gut structure and function during the critical weaning transition.

The intestinal mucosal barrier constitutes a crucial defence system that protects the host against pathogen invasion and maintains gut homeostasis. This barrier comprises mechanical, immune, microbial, and chemical components that act synergistically to preserve intestinal integrity [53]. Within this system, the colonic microbiota, with a population of up to 10^13^ microbes, plays central roles in immune modulation, disease prevention, and metabolic regulation, thereby shaping both local and systemic health [54, 55]. Dietary interventions are known to remodel the gut microbial composition in a dose-dependent manner and shifts in specific taxa may have profound implications on intestinal physiology [56]. Members of the Ruminococcaceae and Lachnospiraceae families, along with the *Bacteroidales *NF3B31 group, are known for their ability to degrade complex carbohydrates and produce SCFAs, supporting energy harvest and epithelial function [57]. *Prevotella* species, including *Prevotella copri*, metabolize dietary fibre and amino acids into SCFAs and BSCFAs such as isobutyrate and isovalerate, linking microbial metabolism to barrier protection and host energy balance [58]. Similarly, *Succinatvibrio* contributes to the metabolism of succinate, an important precursor of propionate [59]. At higher supplementation levels, enrichment of *Alloprevotella* may facilitate carbohydrate fermentation and mucosal health, whereas *Faecalibacterium* is a well-established butyrate producer with anti-inflammatory properties that reinforces barrier integrity [60]. The *Eubacterium coprostanoligenes* group has also been implicated in cholesterol metabolism and intestinal homeostasis [61]. Collectively, these dose-dependent shifts in microbial abundance suggest that sodium isobutyrate may selectively promote beneficial taxa with specialized metabolic functions, thereby enhancing SCFA production, immune regulation, and epithelial protection during the vulnerable weaning transition.

SCFAs, indole derivatives, and bile acids are key metabolites produced by intestinal microbial fermentation [62]. Previous studies have demonstrated that dietary supplementation with SCFAs elevates intestinal acetate and butyrate levels in piglets [31, 63, 64]. In the present study, supplementation with sodium isobutyrate modulated the composition of the intestinal microbiota and enhanced colonic SCFA concentrations in a dose-dependent manner, thereby contributing to the maintenance of intestinal homeostasis. Indole-3-lactic acid, an indole metabolite derived from tryptophan and predominantly produced by *Lactobacillus* species, has been reported to promote gut health and strengthen the intestinal barrier [65–67]. Likewise, 3-hydroxybutyric acid (a ketone body generated from fatty acid metabolism in the liver) has been implicated in delaying the ageing process, reducing oxidative stress, improving bone metabolism in osteoporosis, and alleviating symptoms of ulcerative colitis [68–70]. Methylimidazoleacetic acid, which is functionally related to acetate, may synergistically contribute to intestinal homeostasis [71]. DL-Arginine is essential for neonatal growth and is commonly used as a protein supplement to enhance muscle growth and repair [72, 73]. Collectively, our findings indicate that dietary sodium isobutyrate supplementation beneficially modulates the gut microbiota, increases the generation of favourable metabolites such as SCFAs and 3-hydroxybutyric acid and consequently ameliorates diarrhoea in weaned piglets.

Transcriptomic profiling suggested that sodium isobutyrate supplementation may influence pathways related to protein digestion and absorption as well as the PI3K-Akt signalling cascade, which is consistent with its potential role in supporting nutrient utilization. The PI3K-Akt pathway is broadly regulated by upstream factors such as Toll-like receptors (TLRs), GPCRs, and DDIT4 and has been implicated in epithelial survival, proliferation, and metabolic regulation [74]. Consistent with these observations, the expression of genes related to barrier function and inflammation was also altered. In particular, the expression of genes encoding TJ proteins (CLDN1, OCLN, and ZO-1) increased, whereas the expression of *TSG-6*, *DDIT4*, and *ISG15* were altered in ways that may reflect anti-inflammatory and protective responses. TSG-6 has been described as an anti-inflammatory mediator with therapeutic potential in colitis [75], whereas DDIT4 and ISG15 are stress- and inflammation-related factors [76, 77]. These findings, while preliminary, support the notion that sodium isobutyrate may help preserve epithelial integrity and attenuate inflammatory signalling. Nevertheless, direct functional assays will be needed to confirm whether these changes in the transcriptome and expression levels translate to measurable improvements in barrier function.

The mechanical barrier of the intestine is formed primarily by epithelial cells, intercellular TJs, and the brush border lipid bilayer, together constituting a physical defence that prevents microbial translocation and maintains host homeostasis [78]. During weaning, barrier disruption often leads to increased permeability, with serum biomarkers such as D-LA and DAO serving as widely accepted indicators of barrier integrity [79, 80]. Since LPS derived from Gram-negative bacteria can enter the circulation when the intestinal barrier is impaired, serum LPS levels were measured to assess intestinal barrier integrity [13]. In parallel, TJ proteins play central roles in regulating paracellular transport, and their dysregulation has been implicated in intestinal disorders. Moreover, mucins such as MUC1 and MUC2 contribute to the mucus layer, a complementary component of the barrier [81]. Butyrate and related SCFAs have been reported to reinforce epithelial integrity by enhancing TJ protein expression and mucin production [82, 83]. In this context, the observed modulation of serum biomarkers and TJ-related genes following sodium isobutyrate supplementation suggests that this compound may contribute to improved epithelial barrier function during the vulnerable weaning period. These effects are consistent with the established protective effects of SCFAs on intestinal permeability and epithelial health. However, a limitation of this study is that owing to experimental constraints, the barrier function was inferred only from indirect indicators. Future studies should incorporate functional permeability assays to more robustly validate the beneficial effects of sodium isobutyrate on colonic barrier integrity.

SCFAs, produced by the gut microbiota, serve as essential mediators of host-microbiota interactions and are key regulators of intestinal microbial homeostasis [6, 26]. By functioning as both microbial metabolites and host signalling molecules, SCFAs bridge microbial activity and host immune responses [84]. The biological effects of SCFAs are mediated primarily through two mechanisms: inhibiting histone deacetylase (HDAC) activity, thereby modulating gene transcription, and acting as ligands for GPCRs, which trigger downstream signalling pathways that regulate inflammatory cytokines and enhance intestinal barrier and immune function [85, 86]. The main SCFA-activated GPCRs include GPR41, GPR43, and GPR109A. Previous studies have shown that SCFAs, particularly butyrate, can activate GPR109A to strengthen barrier integrity [87], whereas acetate and propionate have been reported to exert anti-inflammatory effects through GPR41 and GPR43 activation [88, 89]. On the basis of these established functions, the beneficial effects of sodium isobutyrate on colonic barrier integrity may be partly derived from its ability to modulate the gut microbiota and enhance SCFA-mediated GPCR activation. This mechanism provides a plausible link between microbial metabolism and improved barrier function in weaned piglets.

At the molecular level, inflammatory pathway activation is largely regulated by TLRs [90]. These receptors contain a conserved intracellular Toll/interleukin-1 receptor (TIR) domain that mediates signal transduction [91]. As pivotal elements of the innate immune system, TLRs act as pattern recognition receptors (PRRs) that identify microbe-associated molecular patterns and trigger immune activation [92]. The TIR domain facilitates the recruitment of adaptor proteins, notably MyD88, which subsequently transmit downstream signalling events leading to the activation of transcription factors such as NF-κB and the induction of proinflammatory cytokines [93]. Among the TLRs, TLR4 was the first to be identified in mammals and plays a pivotal role in inflammation, including the upregulation of proinflammatory mediators and activation of the inflammasome [94]. Previous research has demonstrated that acetate and butyrate alleviate inflammation by suppressing the TLR4 signalling pathway [95, 96]. Consistent with these findings, our study examined the protein expression of key components of the TLR4 signalling axis. Supplementation with 1,000 mg/kg sodium isobutyrate was associated with lower colonic expression of TLR4, MyD88, and NF-κB, along with reduced levels of downstream inflammatory mediators, including IL-1β, IL-18, TNF-α, and NLRP3. These findings indicate that sodium isobutyrate may mitigate weaning-induced intestinal inflammation by regulating the TLR4/MyD88/NF-κB pathway and balancing inflammatory cytokine expression.

## Conclusion

In summary, dietary supplementation with sodium isobutyrate at appropriate dosages (500, 1,000, and 2,000 mg/kg) significantly improved the growth performance of weaned piglets, with the most effective results achieved at 1,000 mg/kg. These benefits were associated with improved colonic barrier-related markers; an altered gut microbiota composition; increased levels of SCFAs, indole-3-lactic acid, and 3-hydroxybutyrate; activation of the GPR109A receptor; and suppression of the TLR4/MyD88/NF-κB signalling pathway. Transcriptomic and PCR analyses suggested that sodium isobutyrate is involved in pathways related to protein digestion and absorption and PI3K-Akt signalling, particularly through the upregulation of TSG-6 and downregulation of ISG15 and DDIT4. Collectively, these findings provide novel insights into the regulatory role of sodium isobutyrate in intestinal health and immune function and lay a foundation for future studies to elucidate its underlying molecular mechanisms and practical applications in animal production.

## Supplementary Information

Additional file 1: Fig. S1. Metabolic production of isobutyrate and isovalerate by *Bacillus siamensis*. Fig. S2. Effects of isobutyrate, isovalerate and mixed acids on the circle of inhibition of pathogenic bacteria. Table S1. Effects of short-chain fatty acids and sodium salts on MIC of pathogenic bacteria. Table S2. Primers used for mRNA expression analysis via RT-qPCR. Table S3. Primary antibodies. Table S4. PERMANOVA pairwise comparison results.

Additional file 2. The gel and blot images.

## Data Availability

The data used to support the findings of this study are available from the corresponding authors upon request.

## Abbreviations

ADG: Average daily gain
ALB: Albumin
ALT: Alanine aminotransferase
ANOVA: Analysis of variance
AST: Aspartate aminotransferase
BCA: Bicinchoninic acid
BH: Benjamini-Hochberg
BSCFAs: Branched short-chain fatty acids
BUN: Blood urea nitrogen
CAT: Catalase
CF: Crude fibre
CHOL: Total cholesterol
CP: Crude protein
CREA: Creatinine
EE: Ether extract
ELISA: Enzyme-linked immunosorbent assay
FCR: Feed conversion ratio
FPKM: Fragments per kilobase of transcript per million mapped reads
GLOB: Globulin
GLU: Glucose
GPCRs: G protein-coupled receptors
GSH-Px: Glutathione peroxidase
HDAC: Histone deacetylase
HDL: High-density lipoprotein
HRP: Horseradish peroxidase
IL-1β: Interleukin-1β
IL-10: Interleukin-10
IgA: Immunoglobulin A
IgG: Immunoglobulin G
IgM: Immunoglobulin M
KEGG: Kyoto Encyclopedia of Genes and Genomes
LDL: Low-density lipoprotein
MDA: Malondialdehyde
mRNA: Messenger RNA
OM: Organic matter
OTUs: Operational taxonomic units
PLS-DA: Partial least squares discriminant analysis
PVDF: Polyvinylidene difluoride
SCFAs: Short-chain fatty acids
SIgA: Secretory immunoglobulin A
T-SOD: Total superoxide dismutase
SDS-PAGE: SDS–polyacrylamide gel electrophoresis
T-AOC: Total antioxidant capacity
TBA: Total bile acid
TBIL: Total bilirubin
TG: Triglycerides
TJ: Tight junction
TLRs: Toll-like receptors
TIR: Toll/IL-1 receptor
TNF-α: Tumor necrosis factor-α
TP: Total protein
VIP: Variable importance in projection
