# Supplementary material for: Dietary supplementation with sodium isobutyrate enhances growth performance and colonic barrier function in weaned piglets via microbiota-metabolite-host interactions
Source: J Anim Sci Biotechnol. 2025 Dec 8;16:168. doi: 10.1186/s40104-025-01310-w (PMC12683800; doi:10.1186/s40104-025-01310-w)
Supplement: Supplementary file 1 — Additional file 1: Fig. S1. Metabolic production of isobutyrate and isovalerate by Bacillus siamensis. Fig. S2. Effects of isobutyrate, isovalerate and mixed acids on the circle of inhibition of pathogenic bacteria. Table S1. Effects of short-chain fatty acids and sodium salts on MIC of pathogenic bacteria. Table S2. Primers used for mRNA expression analysis via RT-qPCR. Table S3. Primary antibodies. Table S4. PERMANOVA pairwise comparison results. [file 40104_2025_1310_MOESM1_ESM.docx]

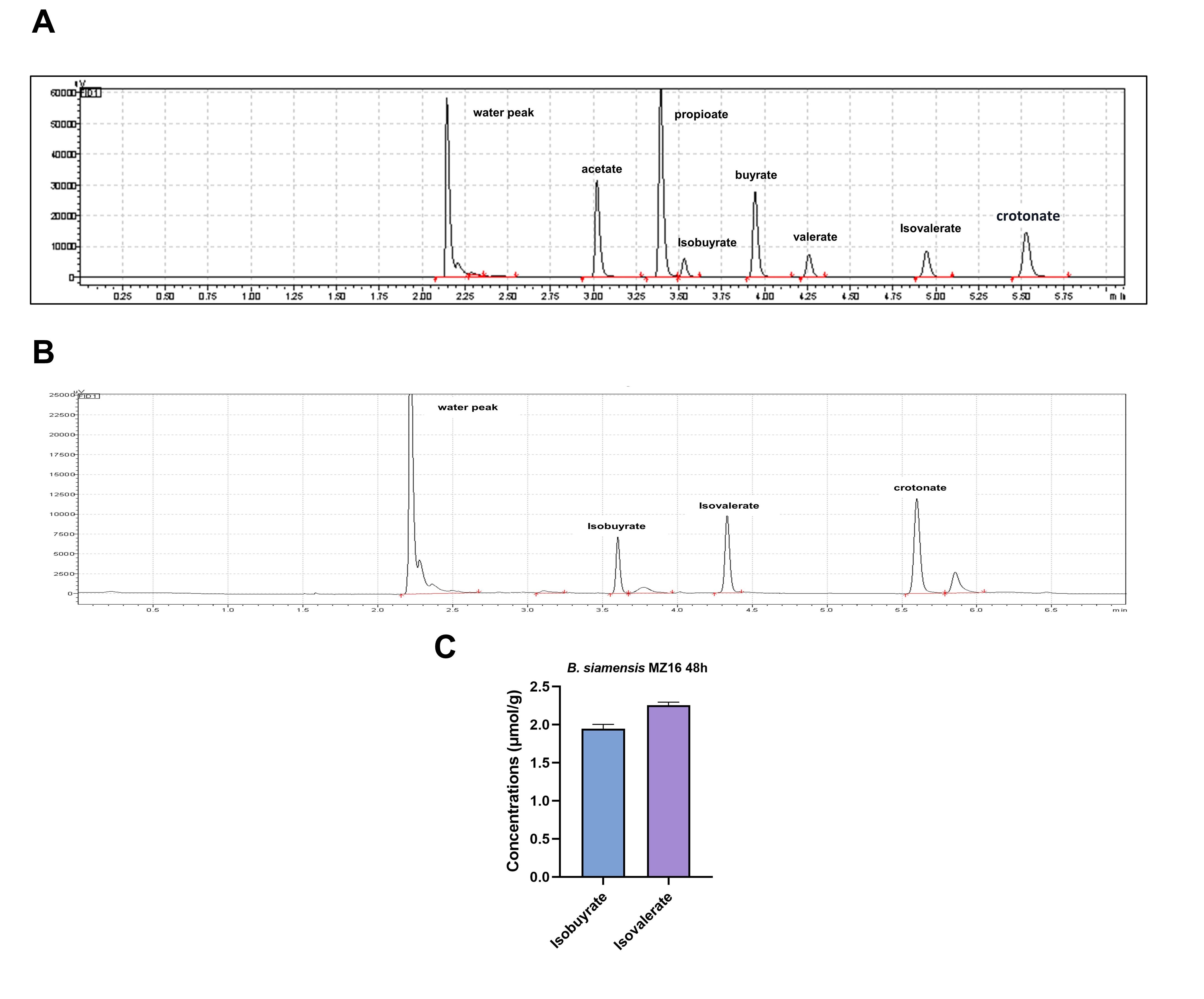


**Figure S1.** Metabolic production of isobutyrate and isovalerate by *Bacillus siamensis*. (*n* = 3)


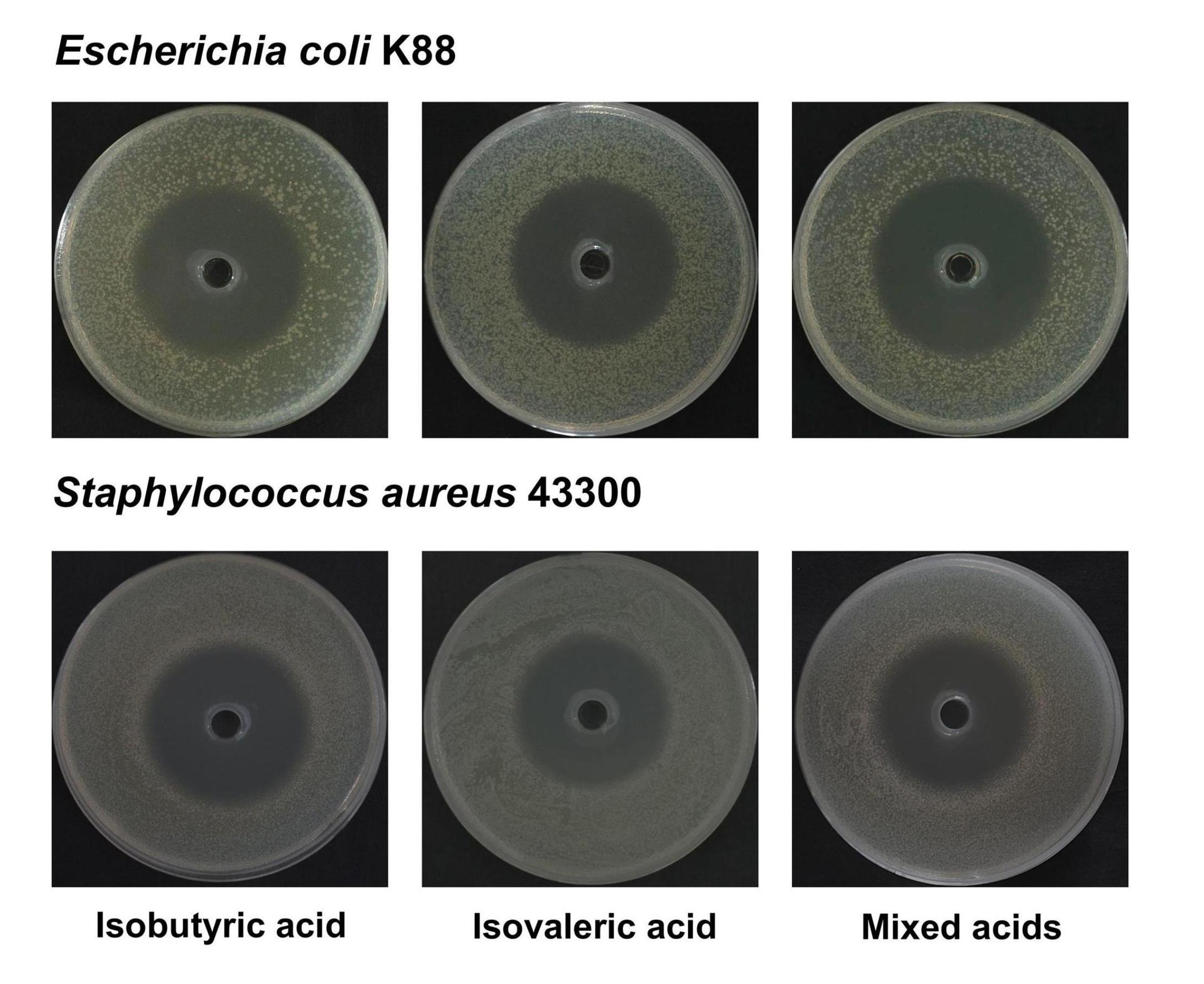


**Figure S2.** Effect of isobutyrate, isovalerate and mixed acids on the circle of inhibition of pathogenic bacteria. (*n* = 3)

| Organic acid  Pathogen | Isobutyric acid | Isovaleric acid | Mixed acids |
| --- | --- | --- | --- |
| *Escherichia coli K88* | 45.82cm | 33.18cm | 37.30cm |
| *Staphylococcus aureus 43300* | 37.11cm | 35.80cm | 35.65cm |

**Table S1.** Effect of short-chain fatty acids and sodium salts on MIC of pathogenic bacteria. (*n* = 3)

| SCFAs and sodium salts MIC（mg/ml） | *Escherichia coli 25922* | *Escherichia coli K88* | *Staphylococcus aureus 43300* |
| --- | --- | --- | --- |
| Acetic acid | 0.25 | 0.25 | 0.25 |
| Propanoic acid | 0.25 | 0.25 | 0.25 |
| Butyric acid | 0.0625 | 0.125 | 0.0625 |
| Isobutyric acid | 0.0625 | 0.125 | 0.125 |
| Isovaleric acid | 1 | 1 | 1 |
| Valeric acid | 0.5 | 1 | 1 |
| Sodium butyrate | 8 | 8 | 16 |
| Sodium isobutyrate | 8 | 8 | 32 |

**Table S2.** Primers used for mRNA expression analysis via RT‒qPCR.

| ***Gene names*** | ***Forward Primer (5’ to 3’)*** | ***Reverse Primer (5’ to 3’)*** |
| --- | --- | --- |
| Pig *TSG6* | AGGCGAAAGCGGTGTGTG  AATAC | ACCCAGCAGCACAGACATGAAATC |
| Pig *DDIT4* | TCCTCCTCTTCGTCTTCGTCCTTG | AGCGACCGAGCCCTTCTTCC |
| Pig *ISG15* | GGTCGCAGCAACGCCTATGAG | CGAAAGTCAGCCAGAACTGGTCAG |
| Pig *ZO-1* | TCAAGGTCTGCCGAGACAAC | ATCACAGTGTGGTAAGCGCA  ATCACAGTGTGGTAAGCGCA  ATCACAGTGTGGTAAGCGCA |
| Pig *Occludin* | TTCATTGCTGCATTGGTGAT | ACCATCACACCCAGGATAGC |
| Pig *Claudin-1* | ATGACCCCAGTCAATGCCAG | CAAAGTAGGGCACCTCCCAG |

**Table S3.** Primary antibodies.

| Antibody | Cat. No | Concentration | Supplier |
| --- | --- | --- | --- |
| β-actin | AC026 | WB:1:10000 | ABclonal,Hubei, China |
| GPR41 | A12636 | WB:1:1000 | ABclonal,Hubei, China |
| GPR43 | A18592 | WB:1:1000 | ABclonal,Hubei, China |
| GPR109A | A15611 | WB:1:1000  IHC-P:1:100 | ABclonal,Hubei, China |
| Occludin | A2601 | WB:1:1000 | ABclonal,Hubei, China |
| Claudin1 | A11530 | WB:1:1000  IHC-P:1:100 | ABclonal,Hubei, China |
| ZO-1 | A25306 | WB:1:1000 | ABclonal,Hubei, China |
| MUC-2 | GB120002-100 | IHC-P:1:500 | Servicebio, Hubei,China |
| TLR4 | WL00196 | WB:1:500 | Wanlei Life Sciences,Liaoning,China |
| MYD88 | WL02494 | WB:1:1000 | Wanlei Life Sciences,Liaoning,China |
| NF-κB | WL01273b | WB:1:1000 | Wanlei Life Sciences,Liaoning,China |
| p-NF-κB | WL02169 | WB:1:1000 | Wanlei Life Sciences,Liaoning,China |

**Table S4.** PERMANOVA pairwise comparison results.

| Pairs (Group comparison) | *R*² | *P*-value | *P*.adjusted |
| --- | --- | --- | --- |
| 0 vs 500 | 0.1136 | 0.181 | 0.181 |
| 0 vs 1000 | 0.1605 | 0.001 | 0.0033 |
| 0 vs 2000 | 0.1545 | 0.023 | 0.0256 |
| 0 vs 4000 | 0.1378 | 0.015 | 0.0188 |
| 500 vs 1000 | 0.2474 | 0.001 | 0.0033 |
| 500 vs 2000 | 0.2704 | 0.006 | 0.0100 |
| 500 vs 4000 | 0.1792 | 0.004 | 0.0080 |
| 1000 vs 2000 | 0.2476 | 0.002 | 0.0050 |
| 1000 vs 4000 | 0.2715 | 0.001 | 0.0033 |
| 2000 vs 4000 | 0.1617 | 0.014 | 0.0188 |
