# Supplementary figures and images for: Dietary supplementation with sodium isobutyrate enhances growth performance and colonic barrier function in weaned piglets via microbiota-metabolite-host interactions
Source: J Anim Sci Biotechnol. 2025 Dec 8;16:168. doi: 10.1186/s40104-025-01310-w (PMC12683800; doi:10.1186/s40104-025-01310-w)

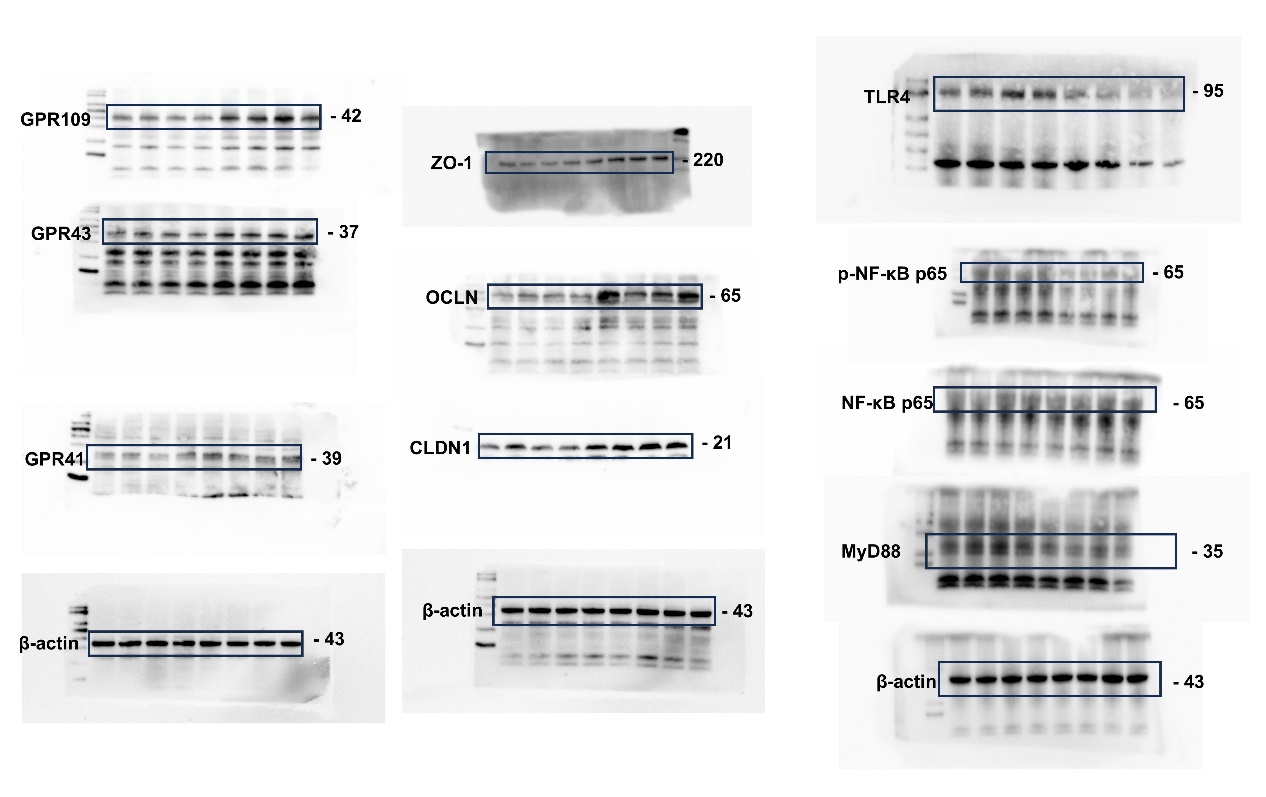


**Figure.** The gels and blots images.

Supplement: Supplementary file 2 — Additional file 2. The gel and blot images. [file 40104_2025_1310_MOESM2_ESM.docx]
